# The Synergistic Protective Effect of γ-Oryzanol (OZ) and N-Acetylcysteine (NAC) against Experimentally Induced NAFLD in Rats Entails Hypoglycemic, Antioxidant, and PPARα Stimulatory Effects

**DOI:** 10.3390/nu15010106

**Published:** 2022-12-26

**Authors:** Ashwag H. Alwadani, Soheir A. Almasri, Amal A. Aloud, Nawal A. Albadr, Ghedeir M. Alshammari, Mohammed Abdo Yahya

**Affiliations:** 1 Department of of Food Science and Nutrition, College of Food and Agricultural Sciences, King Saud University, Riyadh 11451, Saudi Arabia; 2 Department of Home Economics, University College in Farasan, Jazan University, Jazan 54943, Saudi Arabia

**Keywords:** NAFLD, γ-Oryzanol, N-acetylcysteine, hypoglycemic, antioxidant, PPARα

## Abstract

This study estimated that the combined effect of γ-Oryzanol and N-acetylcysteine (NAC) against high-fat diet (HFD)-induced non-alcoholic fatty liver disease (NAFLD) in rats also estimated some of their mechanisms of action. Adult male rats were divided into seven groups (n = 8 each) as control, control + NAC, control + γ-Oryzanol, HFD, HFD + NAC, HFD + γ-Oryzanol, and HFD + NAC + γ-Oryzanol. NAC was administered orally at a final concentration of 200 mg/kg, whereas γ-Oryzanol was added to diets at a concentration of 0.16. All treatments were conducted for 17 weeks and daily. Both NAC and γ-Oryzanol were able to reduce final body weights, fat weights, fasting glucose, fasting insulin, serum, and serum levels of liver function enzymes as well as the inflammatory markers such as tumor necrosis factor-α (TNF-α), interleukine-6 (IL-6), and leptin in HFD-fed rats. They also improved hepatic structure and glucose tolerance, increased adiponectin levels, and reduced serum and hepatic levels of triglycerides (TGs) and cholesterol (CHOL) in these rats. These effects were concomitant with a reduction in the hepatic levels of lipid peroxides (MDA) and serum levels of LDL-C, but also with an increment in the hepatic levels of superoxide dismutase (SOD) and glutathione (GSH). Interestingly, only treatment with γ-Oryzanol stimulated the mRNA levels of proliferator-activated receptor alpha (PPARα) and carnitine palmitoyltransferase 1 (CPT1) in the liver and white adipose tissue (WAT) of rats. Of note, the combination therapy of both drugs resulted in maximum effects and restored almost normal liver structure and basal levels of all the above-mentioned metabolic parameters. In conclusion, a combination therapy of γ-Oryzanol and NAC is an effective therapy to treat NAFLD, which can act via several mechanisms on the liver and adipose tissue.

## 1. Introduction

Non-alcoholic fatty liver disease (NAFLD) is a common liver disease that is associated with obesity, insulin resistance (IR), and diabetes mellitus (DM) and is characterized by excessive lipid accumulation in the liver [1]. The severity of the diseases ranges from simple steatosis, which rapidly progresses into more advanced stages, including non-alcoholic steatohepatitis (NASH), fibrosis, apoptosis, and hepatic carcinoma [2]. To date, the precise mechanism responsible for the development of NAFLD and NASH is still not completely understood, but was identified as a crosstalk mechanism that includes numerous organs—including the liver, adipose tissue, and muscle—and involves hormonal disturbances, IR, and impaired adipose [3].

Hepatic steatosis and insulin resistance, hyperlipidemia, fasting hyperglycemia, and hepatocyte damage are clinical features in patients and animals with NAFLD. However, increased de novo lipogenesis and gluconeogenesis, overproduction of reactive oxygen species (ROS) and inflammatory cytokines, as well as the associated oxidative stress responses and inflammation, are major molecular mechanisms responsible for the progression of steatosis to NASH [4,5]. In this regard, it was demonstrated that the accumulation of free fatty acids (FFAs) in the liver is the major mechanism responsible for lipotoxicity and oxidative stress responses in NAFLD subjects and animals [4,6]. Major resources of these FFAs are the adipose tissue (in response to IR), as well as stimulated synthesis from high glucose and sucrose levels [3,4,6,7]. In addition, this situation is an exaggerated influx of inflammatory cytokines from this adipose tissue, which can trigger Kupfer cell activation, leukocyte recruitment, and ROS production, thus creating a vicious cycle of inflammation/oxidative stress [7,8].

The peroxisome proliferator-activated receptor alpha (PPARα) is the best-known anti-obesity transcription factor in the adipose tissue and liver [9]. Stimulation of PPARα occurs during the fasting state, where it suppresses triglycerides (TGs) synthesis in these tissues by stimulating FA (β) oxidation through increasing the expression of L-carnitine palmitoyl-transferases (CPT1/2) and increasing the mitochondria uptake of FFAs [10,11]. Additionally, PPAR inhibits nuclear factor-kappa-beta (NF-B), which has anti-inflammatory properties [12]. PPARα is significantly depleted in animals and humans with NAFLD [13,14,15]. However, the protective effect of PPARα against obesity and NAFLD has been demonstrated in both human and animal studies, effects that were attributed to their hypolipidemic, hypoglycemic, and anti-inflammatory effects, as well as their ability to improve insulin sensitivity [10,11,15,16,17,18,19]. Furthermore, PPARα-deficient mice suffered from obesity, and their livers showed steatosis, inflammation, oxidative stress, and apoptosis [11,20].

On the other hand, available drugs for NAFLD are those with hypolipidemic effects (e.g., statins) [21]. Unfortunately, these drugs are associated with adverse effects [22]. Accumulating studies have shown encouraging effects of antioxidants in experimental animals, which were also less successful in humans [22]. Currently, more focus is given to plant flavonoids in treating NAFLD due to their multi-pharmacological effects, including potent antioxidants, anti-inflammatory, hypoglycemic, hypolipidemic, and anti-obesity effects [23,24]. Furthermore, flavonoids have shown promising results as an alternative therapy for NAFLD in humans [25,26]. Therefore, searching for new novel drugs to prevent, slow down the progression of, and treat NAFLD is a hot topic [24].

Rice bran (RB), the product of rice milling, is known for its well-reported protective effect against systemic and metabolic disorders due to its antioxidant, anti-inflammatory, anti-obesity hypolipidemic, and hypoglycemic pharmacological properties [27]. These health benefits were attributed to its high content of flavonoids and vitamins [27]. In this context, RB reduced weight gain and adipocyte size, decreased cytokine production from the adipose tissue, improved lipid profile and glycemic index, and protected against hepatic steatosis in DM, diet-induced, and ovariectomized animal models [28,29,30]. In the same way, RB reduced glycemic index, fasting glucose, hemoglobin A1c (HbA1c), cholesterol (CHOL), and TGs levels in diabetic and hyperlipidemic patients [31,32,33]. Gamm-Oryzanol (γ-Oryzanol; C_40_H_58_O_4_) is a major flavonoid found in RB (20%) as a steryl ferulate that is a mixture of ferulic acid esters and triterpene alcohols [34]. As in RB, γ-Oryzanol can attenuate hepatic steatosis and metabolic abnormalities due to its antioxidant, hypolipidemic, anti-diabetic, anti-inflammatory, and anti-obesity effects [35,36,37]. Recently, the protective effect of γ-Oryzanol against NAFLD has been documented in rodents fed a high-fat diet (HFD) and sucrose, effects which were associated with reducing hyperglycemia, attenuating hyperlipidemia and suppressing hepatic oxidative stress and inflammation [38,39].

Despite these reports, the precise molecular mechanism by which γ-Oryzanol affords these anti-obesity and hepatic protective effects is still unknown. In this study, we assumed that chronic treatment of γ-Oryzanol could attenuate HFD-induced NAFLD in rats by stimulating adipose tissue and liver FAs oxidation through regulating PPARα. In addition, we tested the hepatic and anti-steatotic protective effect of this drug in combination with the common antioxidant, N-acetylcysteine (NAC).

## 2. Materials and Methods

### 2.1. Animals

Fifty-six male rats (Wistar species; 10 weeks old, weighing 150 ± 20 g) were supplied by the Experimental Animal Care Center at King Saud University, Riyadh, Saudi Arabia. All protocols conducted on these animals were approved by the Research Ethics Committee (Ethics Reference no. KSU-SE-22-45), King Saud University, Riyadh, Saudi Arabia. All the experimental animal rats were housed in plastic cages (4 rats/cage) at fixed ambient conditions (22 ± 5 °C, 12 h light/dark cycle, humidity 60–64%) throughout the experiment.

### 2.2. Drugs and Experimental Diets

Both standard diet (STD) (Cat. No. Teklad 2014, Envigo, Indianapolis, IN, USA) and HFD (Cat. No. D12451 Research Diets, New Brunswick, NJ, USA) were purchased commercially. These diets provide a total energy of 2.9 kcal/g (13% fat) and 4.73 kcal/g (45% fat), respectively. This HFD diet has induced obesity and NAFLD in rats if chronically consumed for more than 12 weeks (Marques et al., 2015). NAC (Cat. No. A7250) and γ-Oryzanol (Cat. No. 1479202) were purchased from Sigma Aldrich, Louis, MO, USA. NAC was dissolved in 5% carboxymethyl cellulose (CMC) to the desired concentration. However, γ-Oryzanol was added to both STD and HFD at a percent of 0.16% (*w*/*w*). For simplicity, these diets were further named STD-ORL and HFD-ORL

### 2.3. Experimental Design

The animals were adapted for 1 week and then randomly segregated into 7 groups (n = 8 each) (1) STD group: Fed STD and orally treated 5% CMC as a vehicle; (2) STD + NAC-treated rats: fed STD and orally administered NAC (200 mg/kg) dissolved in 5% CMC. (3) STD-ORL-fed rats: fed STD containing 0.16% γ-Oryzanol; (4) HFD-fed rats: fed HFD and orally administered 5% CMC, (5) HFD + NAC-treated rats: fed HFD and orally administered NAC solution (200 mg/kg); (6) HFD + OZ-fed rats: fed HFD containing 0.1% γ-Oryzanol; and (7) HFD + NAC + OZ-fed rats: fed HFD containing 0.1% γ-Oryzanol and co-treated with NAC (200 mg/kg). All experiments were continued for 17 weeks. NAC and vehicle were given by gavage. Changes in body weight and food/calorie intake were calculated every 2 weeks. In our preliminary data (not shown), treating the control rats with a combination of OZ and NAC for 4 weeks resulted in significant hypoglycemia in rats which prevented us from giving both treatments to the control rats.

### 2.4. Dose Selection

The regimen and dose of γ-Oryzanol (0.16%) were based on a recent study by Wang et al. [40], who reported a potent ability of this compound to attenuate liver weights, hepatic steatosis, and hyperglycemia in HFD-fed rats. In addition, the dose of NAC (200 mg/kg) was adopted from another study in rats which showed protective potential against HFD-mediated NAFLD [41].

### 2.5. Oral Glucose Tolerance Test (OGTT)

On the last day of the feeding protocol, each rat of every group was fasted for 12 h and then exposed to the OGTT procedure [42,43]. In brief, the rats were orally treated with glucose solution (2 g/kg glucose), and then 0.25 mL EDTA-blood samples were collected from the tail at baseline (0.0 min) and after different time intervals (30, 60, 90, and 120 min). All blood samples were centrifuged at 1100× *g*, and supernatants were directly used to measure plasma glucose (Cat No. 10009582, Cayman chemicals, Ann Arbor, MI, USA) and insulin levels (Cat. No. 589501, Ann Arbor, MI, USA). Furthermore, the IR homeostasis model assessment (HOMA) was calculated according to the following equation: HOMA-IR = ([glucose (mg/dL) × insulin (ng/mL)]/405). All analyses were conducted for n = 8 samples per group.

### 2.6. Blood and Tissue Sampling

Two days after the OGTT, the rats were fasted again for 12 h and then anesthetized with a ketamine/xylazine solution at a ratio of 80:10 mg/mg. One ml of blood was collected from each rat using the cardiac puncture into plain tubes and used to separate the serum (1100× *g*/10 min/room temperature). Euthanasia was achieved by cervical dislocation. In addition, livers were dissected, weighed, and cut into small pieces. White adipose tissue (WAT) pads—including the inguinal, epididymal, peritoneal, and mesenteric—were identified, separated, and weighed. All tissues were then stored at −80 °C until further use. Parts of the liver of each rat were fixed directly in 10% formalin and sent to the pathology lab for further histological analysis.

### 2.7. Biochemical Analysis in the Serum

Levels of aspartate aminotransferase (AST), alanine aminotransferase (ALT), and gamma-glutamyl transpeptidase (GGT) were measured in the serum of each rat using the rats’ special ELISA kit and as per instructions (Cat. No. MBS264975; Cat. No MBS269614; Cat. No. MBS9343646, and MyBioSorces, San Diego, CA, USA; respectively). All analyses were conducted for n = 8 rats/group.

### 2.8. Biochemical Analysis of Lipids of All Fractions

Stool from each rat was collected during the last week using metabolic cages. Lipids were extracted from the livers and stools using the methanol/chloroform method established by Folch et al. [44]. Serum, hepatic, fecal TGs, and CHOL were measured using assay kits (Cat. No. ECCH-100, BioAssay Systems, Hayward, CA, USA and Cat. No. 10009582, Cayman Chemicals, Ann Arbor, MI, USA). Serum levels of FFAs, high-density lipoprotein-cholesterol (HDL-C), and low-density lipoprotein-cholesterol (LDL-C) were measured using the following assay kits (Cat. No. MBS014345, MyBioSource, San Diego, CA, USA, Cat. No. STA-394, cell Biolabs, San Diego, CA, USA Cat. No. 79960; Crystal Chemicals, Houston, TX, USA). All procedures were conducted for n = 8 rats/group.

### 2.9. Biochemical Analysis in the Liver Homogenates

Frozen liver samples were homogenized in isotonic solution and centrifuged at 1200× *g*/10 min/4 °C. The supernatant of each sample was frozen at −80 °C and then used later to measure levels of leptin (Cat. No. ab100773, Abcam, Cambridge, UK), total glutathione (GSH), (Cat. No. MBS265966, MyBiosources, San Diego, CA, USA), adiponectin (Cat. No. ab239421, Abcam, Cambridge, UK), superoxide dismutase (SOD) (Cat. No. MBS036924 MyBiosources, CA, USA), malondialdehyde (MDA) (Cat. No. MBS738685, MyBiosources, San Diego, CA, USA), tumor necrosis factor-alpha (TNF-α) (Cat. No. BMS622, Thermo Fisher, Germany), and interleukine-6 (IL-6) (Cat. No. R6000B R&D System, Minneapolis, MN, USA). All measurements were carried out for 8 samples/groups.

### 2.10. Real-Time PCR

mRNA levels of markers of FA oxidations, including PPARα and CPT I were measured in the white adipose tissue (WAT) and liver of each rat and normalized to those of GAPDH. The primer pair sequence of all these genes was PPARα [NM_0131961; forward: CCTGCCTTCCCTGTGAACT and reverse: ATCTGCTTCAAGTGGGGAGA], CPT-1 [NM_031559; forward: CCGAGCTCAGTGAGGACCTA and reverse: ATCTGTTTGAGGGCTTCGTG]. CPT-2 [NM_031559; forward: GAGCCCCTAGTAGGCCCTTA and reverse: AGGCTTCTGTGCATTGAGGT]. The primer pair sequence of GAPDH was selected based on the study of Yahya et al. [45]. [NM_017008.3; Forward: GAGATCAACGTGTTCCAGTGC and reverse: CTTCCACCACGTAGGGATTC Forward: GAGATCAACGTGTTCCAGTGC and reverse: CTTCCACCACGTAGGGATTC]. This protocol extracted the total RNA using the Qiagen extraction (Cat. No. 74004). The purity of the RNA was determined using the absorbance 260/280. The first-strand cDNA was synthesized using the supplied commercial kit (Cat. No. K1621 ThromoFisher Waltham, MA, USA, respectively). Amplification of mRNA was conducted using the Ssofast Evergreen Supermix kit (Cat. No. 172-5200, BioRad, Hercules, CA, USA) and Bio-Rad qPCR amplification (model CFX96) as instructed by the kit. The following steps were followed for each target: (1) heating (1 cycle/98 °C/30 s), (2) denaturation (40 cycles/98 °C/5 s), (3) annealing (40 cycles/60 °C/5 s), and (4) melting (1 cycle/95 °C/5 s/step). The relative mRNA expression of PPARα was presented after the normalization of GAPDH using the 2ΔΔCT method. All procedures were performed as instructed by the kit manufacturer’s instructions.

### 2.11. Histopathological Evaluation

The livers were dehydrated in xylene and alcohol of decreasing concentrations—i.e., 100%, 90%, and 70%. The tissue was then placed in wax and cut with a microtome into slices of 3–5 µM thickness. All tissue slices were stained with Harris haematoxylin (H)/glacial acetic acid solution, de-stained with 1:400 *v*/*v* HCL/ethanol (70%) solution, and then stained with eosin (E). Further, the tissue slices were then dehydrated with ethanol and xylene. A mounting media was added, and the tissue slice was covered with a coverslip. The next day, all tissue was examined under a light microscope and photographed at 200×.

### 2.12. Statistical Analysis

All data were analyzed using the GraphPad Prism analysis software (version 8, San Diego, CA, USA). The normality of the data will be tested using the Kolmogorov–Smirnov test. The one-way ANOVA test was used for the analysis using Tukey’s test as post hoc (*p* < 0.05). All data were expressed in the results as means ± standard deviation (SD).

## 3. Results

### 3.1. Changes in Calorie Intake, Fat Deposits, and Body Weights

**Figure 1:**
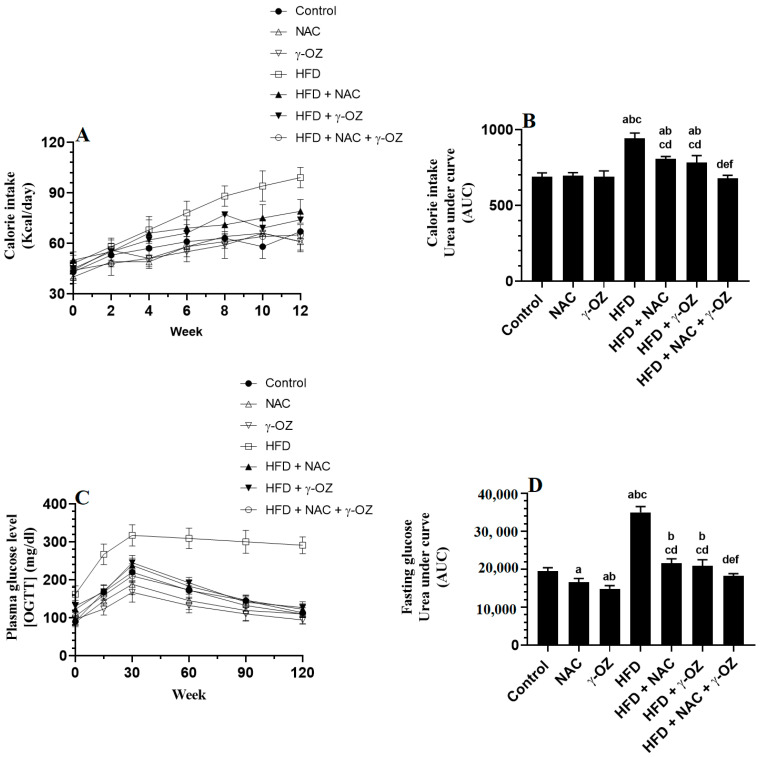
γ-Oryzanol (OZ) and N-acetylcysteine (NAC) and their combination reduces calorie intake and improves oral glucose tolerance test (OGTT) in high-fat-diet (HFD)-fed rats. (**A**): average of weekly calorie intake; (**B**): the corresponding area under the curve (AUC) of the average calorie intake of rats; (**C**): levels of plasma glucose measured before and after the OGTT; and (**D**): AUC of glucose levels measured post-OGTT in (**C**) in all groups of rats. Data are presented as means ± SD (n = 8/group). The value of significance was determined at *p* < 0.05. a: significantly different as compared to the control group; b: significantly different as compared to the NAC-treated control rats; c: significantly different as compared to the γ-Oryzanol-treated rats; and d: significantly different as compared to the HFD-fed group; e: significantly different as compared to the HFD + NAC-treated rats; and f: significantly different as compared to the HFD + γ-Oryzanol-treated rats.

**Figure 2:**
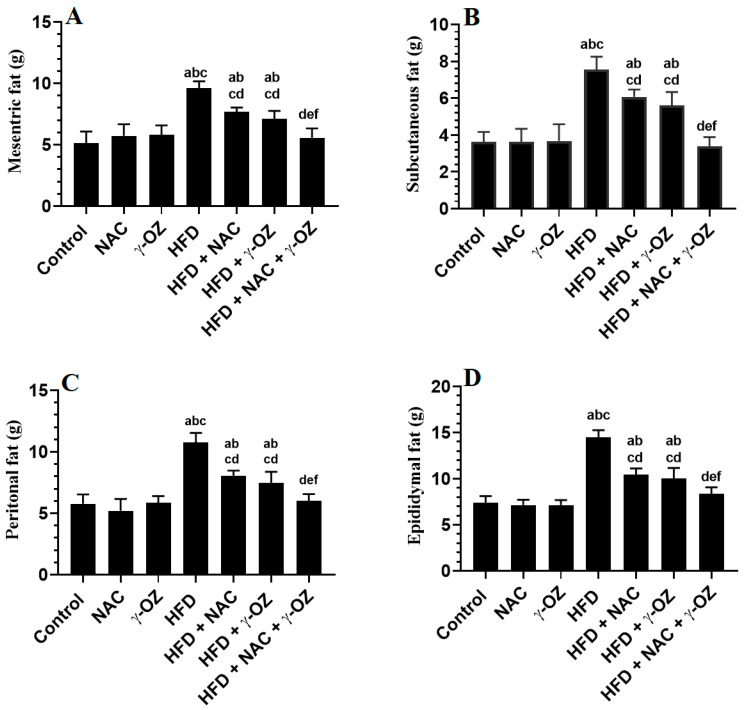
The average weights of various fats in all groups of rats. (**A**): average changes in mesenteric fats; (**B**): average changes in subcutaneous fat; (**C**): average changes in peritoneal fat; and (**D**): average changes in epididymal fat in all groups of rats. Data are presented as means ± SD (n = 8/group). The value of significance was determined at *p* < 0.05. a: significantly different as compared to the control group; b: significantly different as compared to the NAC-treated control rats; c: significantly different as compared to the γ-Oryzanol-treated rats; and d: significantly different as compared to the HFD-fed group; e: significantly different as compared to the HFD + NAC-treated rats; and f: significantly different as compared to the HFD + γ-Oryzanol-treated rats.

Food consumption and calorie intake were measured over the 12 weeks of the study. HFD-fed rats showed a significant and progressive increase in food intake from week 3 to week 12, as compared to control rats, NAC, or γ-Oryzanol (Figure 1A,B). They also had significantly higher weights of mesenteric, peritoneal, subcutaneous, and epidydimal fats, as well as final body weights (Figure 2A–D and Table 1). HFD + NAC, HFD + γ-Oryzanol, and HFD + NAC + γ-Oryzanol showed a significant reduction in the calorie intake during the whole weeks of the study (Figure 1A,B) and all their fat pads, as well as final body weights, were significantly lower than those HFD-fed rats (Figure 2A–D and Table 1). However, the maximum reduction in all these measures was seen in the HFD + NAC + γ-Oryzanol-treated rats, with levels that were not significantly varied with the control rats (Figure 1A,B and Figure 2A–D, and Table 1).

### 3.2. Changes in Fasting Glucose, Insulin, and Glucose Tolerance

HFD-fed rats showed a significant increase in fasting glucose levels and the concentration of glucose measured at 15, 30, 60, 90, and 120 min after the OGTT, as compared to control rats (Figure 1C,D, Table 1). They also had significantly higher levels of insulin and HOMA-IR (Table 1). Serum glucose levels measured during the OGGT, fasting glucose and insulin levels intervals and levels of HOMA-IR were not significantly varied between the control and control + NAC-treated rats but showed a significant reduction in control + γ-Oryzanol-treated rats (Figure 1C,D, Table 1). A significant decrease was seen in HFD + NAC, HFD + γ-Oryzanol, and HFD + NAC + γ-Oryzanol-treated rats (Figure 1C,D, Table 1). Of note, only standard and non-significant glucose curves—as well as normal levels of fasting glucose, fasting insulin, and HOMA-IR, like those seen in the control rats—were seen in HFD + NAC + γ-Oryzanol-treated rats (Figure 1C,D, Table 1).

### 3.3. Changes in SBP and Serum Parameters

Systolic blood pressure (SBP)—as well as serum levels of leptin, TNF-α, IL-6, FFAs, AST, ALT, and GTT—were significantly increased, but serum levels of adiponectin were significantly decreased in the HFD-fed rats as compared to all control groups (Table 1 and Table 2). The levels of all these parameters were significantly reversed in HFD + NAC, HFD + γ-Oryzanol, and HFD + NAC + γ-Oryzanol-treated rats with normal basal levels to be seen in the latter group (Table 1 and Table 2). Interestingly, no significant variations in the majority of all these markers were seen between the control, NAC, and γ-Oryzanol-treated control rats. Only adiponectin showed a significant increase, and FFAs showed a significant decrease in the NAC-treated control rats as compared to the control group (Table 1 and Table 2).

### 3.4. Changes in Serum, Hepatic, and Stool Lipid Profile

Various lipid fractions were measured in the stools, livers, and blood of all groups of rats (Table 3). There was a significant increment in the serum, hepatic, and fecal levels of TGs and GHOL in HFD-fed rats as compared to control rats (Table 3). They also had significantly higher serum levels of LDL-C and hepatic levels of FFAs but low levels of HDL-c (Table 3). A reversal of these lipid levels in the liver and serum was confirmed in the HFD + NAC, HFD + γ-Oryzanol, and HFD + NAC + γ-Oryzanol-treated rats (Table 3). Among all these groups, normal basal hepatic and serum levels of TGs and CHOL, as well as normal serum levels of LDL-C and HDL-c, were seen only in the HFD + NAC + γ-Oryzanol-treated rats (Table 3). However, fecal TGs and CHOL were not significantly varied between the control HFD rats and HFD-fed groups of all treatments, as well as between the STD-fed, NAC, and γ-Oryzanol-treated control rats (Table 3). It remains worth mentioning that serum and hepatic levels of TGs and CHOL, as well as serum levels of LDL-C, were significantly lower, but serum levels of HDL-C were significantly higher in both the NAC and γ-Oryzanol-treated control rats, as compared to control rat-fed the STD (Table 3).

### 3.5. Changes in Hepatic Antioxidant and Inflammatory Markers

**Figure 3:**
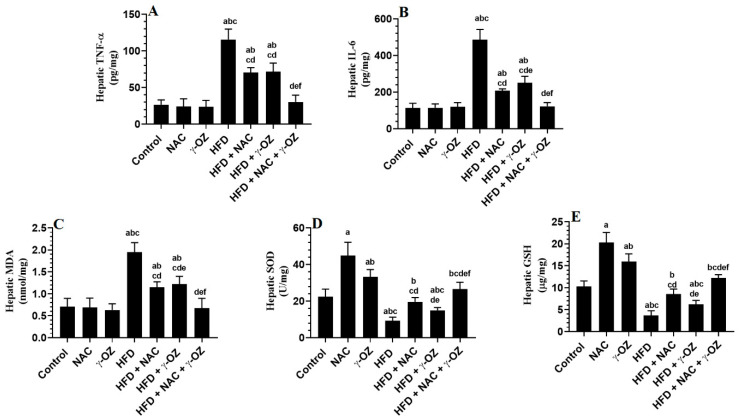
γ-Oryzanol (OZ) and N-acetylcysteine (NAC) and their combination represses inflammation and lipid peroxidation but improve antioxidants in the livers of high-fat-diet (HFD)-fed rats. (**A**): average levels of tumor necrosis factor-α (TNFα); (**B**): average levels of interleukin-6 (IL6); (**C**): average levels of malondialdehyde (MDA); (**D**): average levels of superoxide dismutase (SOD), and (**E**): average levels of total glutathione (GSH) in the livers of all groups of rats. Data are presented as means ± SD (n = 8/group). The value of significance was determined at *p* < 0.05. a: significantly different as compared to the control group; b: significantly different as compared to the NAC-treated control rats; c: significantly different as compared to the γ-Oryzanol-treated rats; and d: significantly different as compared to the HFD-fed group; e: significantly different as compared to the HFD + NAC-treated rats; and f: significantly different as compared to the HFD + γ-Oryzanol-treated rats.

Hepatic levels of TNF-α, IL-6, and MDA were not significantly different, but levels of SOD and GSH were significantly higher in NAC and γ-Oryzanol-treated control when compared to the control group of STD-fed rats (Figure 3). However, HFD-fed rats showed a significant increment in the hepatic levels of TNF-α, IL-6, and MDA and showed a significant decline in the levels of GSH and SOD, as compared to all control groups (Figure 3). A significant reduction in the levels of TNF-α, IL-6, and MDA that is parallel with a significant increase in the levels of SOD and GSH was seen in the livers of HFD + NAC, HFD + γ-Oryzanol, and HFD + NAC + γ-Oryzanol-treated rats, as compared to HFD-fed (Figure 3). In line with all other results, normal basal hepatic levels of all these biochemical endpoints were only seen in the HFD + NAC + γ-Oryzanol-treated rats (Figure 3).

### 3.6. Changes in mRNA Levels of PPARα and CPT-1

**Figure 4:**
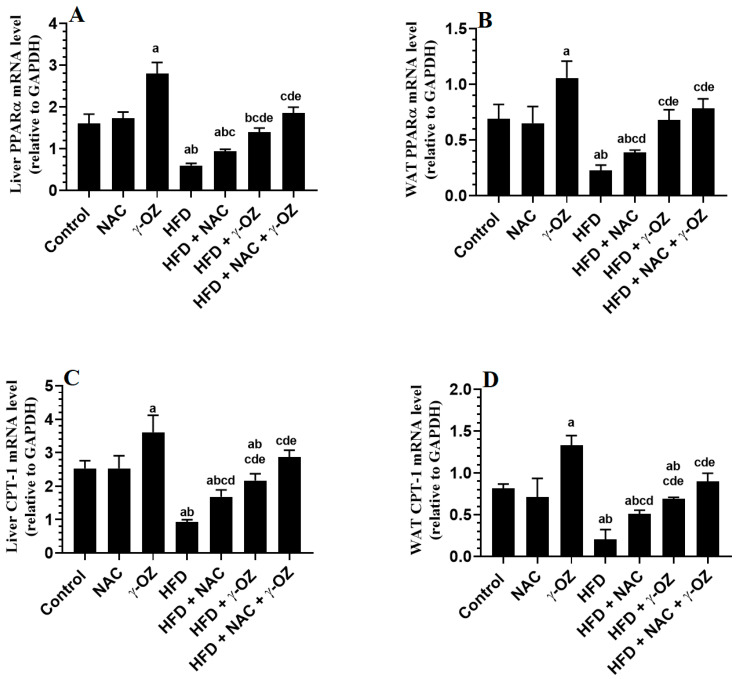
γ-Oryzanol (OZ) and N-acetylcysteine (NAC) and their combination downregulate the transcription of peroxisome proliferator-activated receptor alpha (PPARα) and carnitine palmitoyl transferase 1 (CPT1) in the livers (**A**,**C**) and white adipose tissue (WAT) (**B**,**D**) in all groups of rats. Data are presented as means ± SD (n = 8/group). The value of significance was determined at *p* < 0.05. a: significantly different as compared to the control group; b: significantly different as compared to the NAC-treated control rats; c: significantly different as compared to the γ-Oryzanol-treated rats; and d: significantly different as compared to the HFD-fed group; and e: significantly different as compared to the HFD + NAC-treated rats.

A significant increase in the mRNA levels of PPARα and CPT-1 was seen in γ-Oryzanol-treated control rats as compared to control (STD) rats (Figure 4A–D). No significant variations in the mRNA of these genes were seen between the STD-fed and NAC-treated control rats (Figure 4A–D). Hepatic mRNA levels of PPARα and CPT-1 (Figure 4A,C) and WAT mRNA levels of PPARα and CPT-1 (Figure 4B,D) were significantly decreased in the livers and WAT (Figure 4B,D) of HFD-fed rats as compared to control, γ-Oryzanol, and NAC-treated control rats. On the contrary, hepatic mRNA levels of PPARα and CPT-1 (Figure 4A,C) and WAT mRNA levels of PPARα and CPT-1 (Figure 4B,D) were significantly increased in the livers HFD + NAC, HFD + γ-Oryzanol, and HFD + NAC + γ-Oryzanol-treated rats as compared to HFD-fed rats. The maximum reduction in the expression of both genes was seen in the livers of HFD + NAC + γ-Oryzanol, as compared to HFD + NAC and HFD + γ-Oryzanol-treated rats (Figure 4A–D).

### 3.7. Histopathological Studies

**Figure 5:**
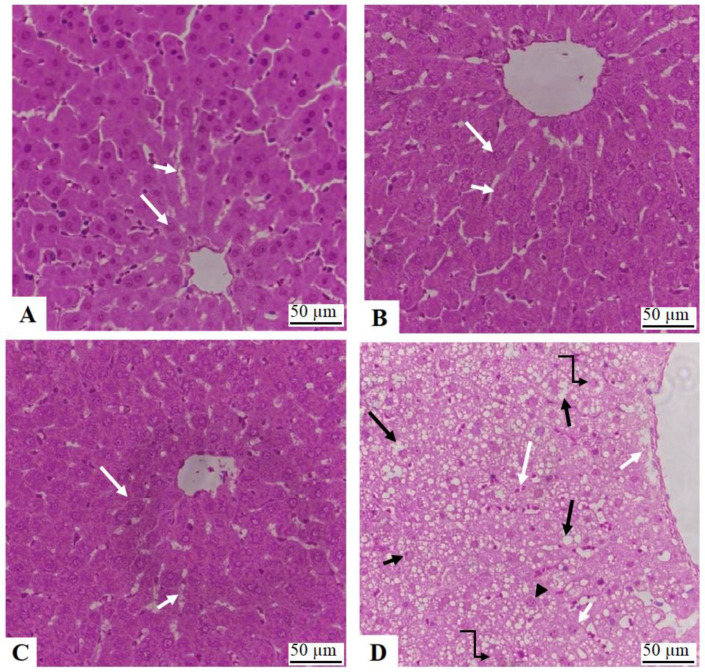
Liver histology sections from control groups and HFD model rats. (**A**–**C**): were taken from control, NAC, and + γ-Oryzanol-treated animals, respectively, and showed normal liver pathology, including normal central vein (CV) in which normally appearing hepatocytes are radiating (long white arrow). In addition, these cells had normally sized sinusoids (short white arrow). (**D**): was taken from an HFD-fed animal and showed a typical image of liver steatosis. The CV was greatly enlarged with evidence of some cell necrosis around it (short white arrow). The majority of the cell cytoplasm was filled with fat droplets (long black arrow). In addition, karyolysis (black arrowhead), pyknosis (short arrow), and karyorrhexis (short black arrow) in a majority of the hepatocytes (curved black arrow) were dominant and in large numbers. Immune cell infiltration was also seen (large white arrow).

**Figure 6:**
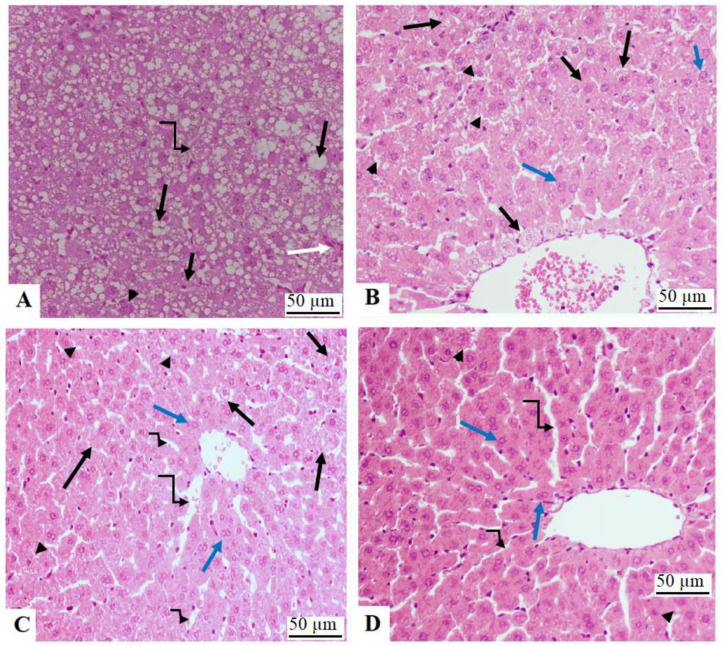
Liver sections from HFD model rats and other HFD treated groups: (**A**): was taken from HFD-fed animals and showed increased fat accumulation in the cell cytoplasm and the appearance of large fat vacuoles (long black arrow). Note the increased number of cells with karyolysis (black arrowhead), pyknosis (short arrow), and karyorrhexis (short black arrow). In addition, immune cell infiltration was evident (white arrow). (**B**,**C**): were taken from HFD + NAC and HFD + γ-Oryzanol-treated animals, respectively, and showed an obvious improvement in liver structure with an increased number of normal cells (Blue arrow) and sinusoids (small curved arrow). However, some pathological findings, including swollen cells (long black arrowhead), cells filled with fat (long large arrow), dilated sinusoids (large, curved arrow), and necrotic cells (very long black arrow), are still seen. (**D**): was taken from an HFD + γ-Oryzanol-treated rat and showed almost normal liver features like the control group. The majority of the cells in the field (long black arrow), as well as the sinusoids (short curve arrow), appeared normal. However, some necrotic cells (black arrowhead) and dilated sinusoids (long curved arrow) are still present but were few.

Livers from control, NAC, and γ-OZ showed normal liver features, including central veins, sinusoids, and hepatocytes (Figure 5A–C). On the other hand, increased cytoplasmic lipid vacuolations with an increased number of cells with karyolysis, pyknosis, and karyorrhexis were seen in the livers of HFD-fed animals (Figure 5D and Figure 6A). HFD co-treated with NAC or γ-OZ, as well as those co-treated with a combined dose of NAC (Figure 6B) and γ-OZ (Figure 6C), as well as those co-treated with both NAC and γ-OZ (Figure 6D) showed improvement in the structure of the hepatocytes with a significant reduction in the number of and size of cytoplasmic vacuoles. In addition, these tissues showed an increased number of normal hepatocytes. Almost normal tissue features were seen in the combined treatment group.

## 4. Discussion

**Figure 7:**
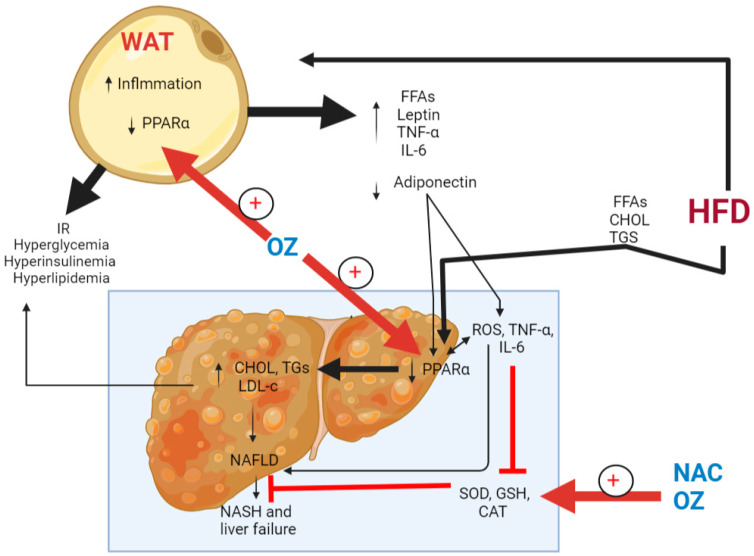
A graphical abstract showing the synergistic mechanistic effect of γ-Oryzanol and N-acetylcysteine (NAC) against high-fat diet (HFD) induced non-fatty liver. In the figure, HFD promotes inflammation and insulin resistance (IR) and downregulates PPARα in the white adipose tissue (WAT). This activates lipogenesis in WAT, which increases the influx of inflammatory cytokines and free fatty acids (FFAs) to the liver. It also increases the release of leptin and lowers those of adiponectin. Fruthermore, the influx of dietary FFAs increases in the liver of rats. As a result of all these factors, the generation of reactive oxygen species (ROS), tumor necrosis factor-α (TNF-α), and interleukin-6 (IL-6) are stimulated in the livers of rats which scavenge major antioxidants such as glutathione (GSH), superoxide dismutase (SOD), and catalase (CAT). In addition, the levels PPARα are decreased, which stimulates the hepatic de novo lipid synthesis and results in hepatic steatosis and non-alcoholic fatty liver disease (NAFLD). In the presence of low antioxidant power and high levels of ROS and inflammatory cytokines, the disease can progress to non-alcoholic steatohepatitis (NASH) and liver failure. NAC inhibits oxidative stress and inflammation by stimulating major antioxidant proteins, CAT, SOD, and GSH. On the other hand, γ-Oryzanol stimulates GSH, SOD, and CAT and stimulates the levels of PPARα and both the liver and WAT.

This study demonstrates that the combination therapy of both γ-Oryzanol and NAC is an effective treatment to ameliorate NAFLD and its metabolic abnormalities (e.g., hyperlipidemia, hyperglycemia, hypertension, and IR) in rats. The novelty of this study also shows a synergistic mechanism by which NAC and γ-Oryzanol collaborate to protect such hepatic and steatosis effects by attenuating HFD-mediated oxidative stress and inflammation. In addition, γ-Oryzanol adds a powerful effect mediated by its potent hypoglycemic effect and its ability to modulate de novo lipogenesis by stimulating PPARα/CPT-1 induced FA oxidation. A full mechanism of action is shown in the graphical abstract (Figure 7).

Increased calorie intake and chronic feeding HFD promote metabolic syndrome (MetS), which is the most known risk factor for the development and progression of NAFLD and NASH [45,46,47]. HFD feeding of the rats of this study resulted in features of metabolic syndrome—including obesity, dyslipidemia, fasting hyperglycemia, hyperinsulinemia, IR, and hypertension—which validate our animal model and support other studies [1,48,49]. On the other hand, individual or combined treatment of both γ-Oryzanol and NAC attenuated all these metabolic disturbances induced by HFD. In addition, they also attenuated hepatic steatosis and reduced hepatic fat accumulation in the model rats. Furthermore, both drugs prevented the gain in fat deposits and significantly reduced the calorie intake and final body weights of these HFD-fed rats. Hence, we have concluded that both NAC and γ-Oryzanol are excellent adipogenic, anti-obesity, and anti-steatotic molecules that can prevent the development and progression of NAFLD. Interestingly, we have shown an exceptional ability of NAC and γ-Oryzanol to reduce glucose, insulin, hepatic and serum levels of TGs, and CHOL, even in control rats that fed the STD, thus confirming their dependent hypoglycemic and hypolipidemic effects.

These findings support others who have found similar hypoglycemic, hypolipidemic, insulin-improving, and anti-steatosis effects of γ-Oryzanol in sucrose, fructose, and HFD-fed rats, possibly due to their effect to reduce fecal lipid excretion (inhibiting intestinal lipase), inhibit gluconeogenesis enzymes (e.g., G6PD), and suppress lipogenic enzymes (e.g., malic enzyme, SREBP-1c, and fatty acid synthase) [40,50,51]. In addition, γ-Oryzanol reduced the body weights of obese rodents and inhibited the differentiation and increase in the size of human cultured adipocytes [50,52]. It also lowered blood pressure and prevented hepatic cirrhosis in hypercholesterolemia-spontaneous hypertensive rats by reversing dyslipidemia [53]. In the same line, NAC has an independent hypolipidemic effect mediated by downregulating the hepatic FAs receptors (D36), SREBP1/2, and PPARγ [54,55]. Treatment with NAC attenuated fasting hyperglycemia, IR, and hypertension and improved peripheral insulin sensitivity in HFD and IR animal models [55,56,57]. It is noteworthy that fecal lipid levels of TGs and CHOL were not significantly different between HFD rats that received the vehicle or the individual or combined treatment of NAC and γ-Oryzanol, thus dissipating their effect on intestinal lipid absorption and contradicting those reported by Francisqueti et al. [39].

Leptin and adiponectin are the two major hormones released from adipose tissue. Leptin is the polyphagia-related hormone that stimulates food intake, whereas adiponectin is an anti-adipogenic hormone that improves insulin signaling and FAs oxidation and suppresses FA oxidation [58,59]. Low levels of adiponectin or adiponectin resistance that is concomitant with a sustained increase in leptin levels were seen in obese and NAFLD animals and subjects [60]. However, higher adiponectin levels protected against NAFLD in rats by modulating glucose and lipid metabolism, as well as insulin signaling, oxidative stress, and inflammation [61]. In this study, we have also seen higher leptin and low adiponectin levels in the sera of HFD-fed rats. Such an increase in leptin levels could explain the increase in food and calorie intake of these rats during the whole period of the study. On the other hand, the reduction in circulatory adiponectin could be considered an extra mechanism that worsens hepatic inflammation, oxidative stress, lipogenesis, and IR [61]. Interestingly, the partial reversal in the levels of these hormones in the HFD rats after γ-Oryzanol or NAC therapy may explain why these rats showed a reduction in food/calorie intake and could partially be attributed to their hepatoprotective effects. It is worth noting that treatments with γ-Oryzanol not only stimulated the release of adiponectin in HFD-fed rats but also in those fed the STD, indicating an interesting mechanism of action. Similarly, γ-Oryzanol also restored the expression and length of adiponectin levels in a stress-induced model of hypoadiponectinemia [62].

On the other hand, the increased WAT lipolysis due to IR is the major mechanism by which NAFLD develops in obese individuals and HFD-fed animals [3,4]. IR is the key player in the process that promotes hepatic oxidative stress and inflammation, mainly by increasing the influx of FFAs and cytokines from the impaired adipose tissue [3,4,6,7]. Antioxidants and anti-inflammatory agents protect against NAFLD and prevent its progression to nonalcoholic steatohepatitis (NASH) [63,64]. Levels of IL-6 and TNF-α, as well as markers of lipid peroxidations and reduced endogenous antioxidants, were estimated in the serum and livers of NAFLD animals and subjects [65]. This study also saw a similar mirror image in the livers of HFD-fed rats. Indeed, NAC and γ-Oryzanol reduced the contents of TNF-α, IL-6, and MDA but stimulated SOD and GSH in the livers of the control and HFD-fed rats. These data indicate individual and independent antioxidant and anti-inflammatory potentials of NAC and γ-Oryzanol. This can be supported by the numerous reports showing the dependent-antioxidant potential of both drugs [52,66].

Indeed, NAC is a precursor of GSH that can prevent tissue damage in a variety of disorders by boosting GSH, scavenging ROS, and suppressing inflammatory cytokine generation. NAC also prevented NAFLD in HFD, cholesterol-fed, methionine–choline-deficient (MCD) animal models by its ability to reduce hepatic lipogenesis and alleviate oxidative stress and inflammation by boosting GSH and antioxidant enzymes [67]. Furthermore, NAC reduced the expression of TNF-α, IL-6, and IL-1β by suppressing NF-κB [68]. Likewise, γ-Oryzanol stimulated hepatic levels of GSH and SOD, CAT, glutathione peroxidase (GPx), and glutathione reductase (GR) and reduced lipid peroxidation in HFD and sucrose-fed animals [50]. Such antioxidant potential of γ-Oryzanol was explained by its exceptional ability to donate hydrogen from its ferulic acid constituent, as well as by its ability to inhibit iron-driven hydroxyl radical formation [50,69,70,71]. Additionally, γ-Oryzanol prevented neuromotor defects and reduced dopamine by suppressing lipid peroxidation and oxidative stress by upregulating SOD, CAT, and glutathione-S-transferase in the *Drosophila melanogaster* model of Parkinson’s disease [72]. Moreover, Islam et al. [73] demonstrated that this molecule could prevent inflammatory colitis in rats by suppressing the transcription of different inflammatory cytokines (i.e., IL-6, IL-1β, TNF-α) and decreasing leukocyte infiltration. A year later, the same authors have shown a potent ability of γ-Oryzanol to suppress NF-κB in LPS-stimulated RAW 264.7 [71].

Nonetheless, PPARα agonists such as fibrate are currently used clinically to treat hyperlipidemia and reduce the risk of CVDs [74]. In general, PPARα can be found in numerous tissues—including the heart, liver, and adipose tissue—where its major function is to stimulate FA oxidation by increasing the expression of many related genes such as CPT1/2 and other uncoupling protein (UCP1-3) [74,75]. PPARα can also stimulate the synthesis of HDL-c and reduce levels of LDL-c [75]. In addition, the activation of the PPARα suppressed hepatic and adipose tissue lipogenesis by inhibiting SREBP1c through the activation of Insig2a [9]. PPARα can also prevent tissue inflammation by suppressing the NF-κB and the synthesis of IL-1β, TNF-α, and IL-6 [76]. PPARα is significantly depleted in the liver, WBC, and adipose tissue of HFD-fed and obese animals or subjects [13,77,78,79]. Pharmacological or genetic overexpressing of PPARα reduced the gain in body weights, decreased serum levels of TGs and CHOL, increased insulin sensitivity, suppressed the increase in adipocyte size, and stimulated hepatic and adipose tissue mitochondrial FAs oxidation by upregulating CTP1, CPT2, and UCP1-3 [12,80,81,82].

Similar to this evidence, mRNA levels of PPARα and CPT1 were significantly decreased in the livers of HFD-fed rats. However, only the treatment with γ-Oryzanol stimulated these genes’ levels in the liver and adipose tissue of HFD-fed rats. They also stimulated the expression of these genes in the livers and adipose tissues of the control rats too. The data suggest that γ-Oryzanol acts as an anti-obesity, antihyperlipidemic, and anti-adipogenic molecule by stimulating PPARα. This finding is novel and shows that Oryzanol may act as a PPARα agonist to treat obesity, hyperlipidemia, and IR in metabolic conditions. However, since NAC does not affect the expression of PPARα, we dissipate this mechanism from its action. The most interesting finding in this study is that co-therapy with both NAC and γ-Oryzanol was the most effective therapy, which completely abolished the above-mentioned metabolic, oxidative stress, and inflammatory markers. This could be due to their synergistic effects, each of which acts by different mechanisms, as discussed above.

## 5. Conclusions

Chronic administration of a combination of NAC and γ-Oryzanol is an effective therapy to prevent NAFLD and other features of metabolic syndrome. This combination synergistically reduces fasting hyperglycemia, hyperlipidemia, and hypertension; improves peripheral and hepatic insulin sensitivity; alleviates hepatic oxidative stress and inflammation; and prevents lipid accumulation in the WAT and liver. However, while both treatments are potent hypoglycemic and antioxidant molecules, γ-Oryzanol stimulates WAT and hepatic FA oxidation by stimulating the PPARα/CPT1 axis. In addition, given the high safety and pre-clinical use of both drugs in humans, these data encourage further subclinical and clinical use of this combination which could present a novel treatment for obesity, IR, and NAFLD.

## 6. Limitations of the Study

Despite our findings, this study still has some limitations. Even our data have shown a stimulatory effect of both NAC and γ-Oryzanol on the hepatic and WAT levels of PPARα, and this finding remains observational and more experiments using PPARα-deficient cells or animals may validate this. In addition, a dose–response, as well as a time-dependent designed study are required to precisely study the metabolic effect of both treatments on liver lipid metabolism. Furthermore, targeting other signaling pathways such as SIRR1 and AMPK—which normally regulate lipid metabolisms, biogenesis, and oxidation and can regulate PPARα—were not studied in this study and should be targeted in a future study to examine the upstream mechanism of action of these drugs.

## Figures and Tables

**Table 1:** γ-Oryzanol (OZ) and N-acetylcysteine (NAC and their combination attenuate the increase in body weights, hyperglycemia, hyperinsulinemia, and the alterations in circulatory adipokines in high-fat-diet (HFD)-fed rats.

Parameter	Control	NAC	γ-OZ	HFD	HFD + NAC	HFD + γ-OZ	HFD + NAC + γ-OZ
Final body weight (g)	489 ± 32	501 ± 35	492 ± 29	642 ± 38 ^abc^	565 ± 32 ^abcd^	541 ± 26.8 ^abcd^	11 ± 26 ^def^
Fasting plasma glucose (mg/dL)	97.2 ± 9.3	88.9 ± 8.4	84 ± 9.5 ^a^	162 ± 15.6 ^abc^	122 ± 11.6 ^abcd^	131 ± 15.1 ^abcd^	104 ± 9.3 ^def^
Fasting insulin levels (ng/mL)	2.7 ± 0.47	3.1 ± 0.52	2.1 ± 0.54 ^a^	6.7 ± 1.2 ^abc^	4.9 ± 1.1 ^abcd^	3.9 ± 0.89 ^abcd^	2.9 ± 0.49 ^def^
HOMA-IR	0.64 ± 0.07	0.68 ± 0.05	0.43 ± 0.02 ^a^	2.61 ± 0.33 ^abc^	1.5 ± 0.35 ^abcd^	1.3 ± 0.36 ^abcd^	0.71 ± 0.69 ^def^
SBP (mmHg)	114 ± 8.7	109 ± 9.1	103 ± 9.7	156 ± 12.8 ^abc^	126 ± 9.1 ^abcd^	129 ± 11.8 ^abcd^	109 ± 9.1 ^def^
Adiponectin (µg/mL)	48.5 ± 5.8	62.6 ± 5.1 ^a^	59.1 ± 4.6 ^a^	22.7 ± 3.6 ^abc^	35.2 ± 5.3 ^abcd^	39.9 ± 4.2 ^abcd^	47.1 ± 5.4 ^bcdef^
Leptin (ng/mL)	18.3 ± 2.9	20.1 ± 3.4	17.6 ± 3.1	45.2 ± 5.9 ^abc^	31.4 ± 4.7 ^abcd^	27.3 ± 3.3 ^abcd^	18.9 ± 2.2 ^def^
Serum FFAs (µmol/L)	552 ± 39	492 ± 48	387 ± 53 ^a^	2002 ± 192 ^abc^	982 ± 83 ^abcd^	821 ± 79 ^abcd^	483 ± 31 ^def^
Serum TNF-α (pg/mL)	178 ± 15.4	161 ± 15.7	173 ± 16.5	332 ± 21.8 ^abc^	219 ± 22.4 ^abcd^	204 ± 19.9 ^abcd^	167 ± 14.2 ^def^
Serum IL-6 (pg/mL)	22.5 ± 3.6	19.4 ± 2.8	22.7 ± 2.6	53.4 ± 4.8 ^abc^	37.5 ± 5.6 ^abcd^	31.2 ± 3.4 ^abcd^	23.2 ± 4.2 ^def^

Data are presented as means ± SD (n = 8/group). The value of significance was determined at *p* < 0.05. a: significantly different as compared to the control group; b: significantly different as compared to the NAC-treated control rats; c: significantly different as compared to the γ-Oryzanol-treated rats; and d: significantly different as compared to the HFD-fed group; e: significantly different as compared to the HFD + NAC-treated rats; and f: significantly different as compared to the HFD + γ-Oryzanol-treated rats.

**Table 2:** γ-Oryzanol (OZ) and N-acetylcysteine (NAC) and their combination reduce liver function enzymes in high-fat-diet (HFD)-fed rats.

Parameter	Control	NAC	γ-OZ	HFD	HFD + NAC	HFD + γ-OZ	HFD + NAC + γ-OZ
ALT (U/L)	31 ± 3.3	35 ± 4.1	28 ± 3.7	78 ± 8.1 ^abc^	52 ± 4.8 ^abcd^	61 ± 6.8 ^abcd^	32 ± 4.3 ^def^
AST (U/L)	43 ± 6.5	48 ± 4.2	46 ± 5.2	112 ± 10.1 ^abc^	71 ± 7.3 ^abcd^	65 ± 6.5 ^abcd^	48 ± 5.4 ^def^
GTT	17 ± 2.1	19 ± 2.7 ^a^	2.1 ± 3.9	72 ± 5.2 ^abc^	41 ± 4.9 ^abcd^	37 ± 5.8 ^abcd^	25 ± 3.5 ^abcdef^

Data are presented as means ± SD (n = 8/group). The value of significance was determined at *p* < 0.05. a: significantly different as compared to the control group; b: significantly different as compared to the NAC-treated control rats; c: significantly different as compared to the γ-Oryzanol-treated rats; and d: significantly different as compared to the HFD-fed group; e: significantly different as compared to the HFD + NAC-treated rats; and f: significantly different as compared to the HFD + γ-Oryzanol-treated rats. AST: aspartate aminotransferase (AST); ALT: alanine aminotransferase; and GTT: gamma-glutamyl transpeptidase.

**Table 3:** γ-Oryzanol (OZ) and N-acetylcysteine (NAC) and their combination improve lipid profile in the serum and livers of high-fat-diet (HFD)-fed rats.

	Parameter	Control	NAC	γ-OZ	HFD	HFD + NAC	HFD + γ-OZ	HFD + NAC + γ-OZ
Serum	TGs (mg/dL)	58 ± 4.6	45 ± 4.9 ^a^	41 ± 4.1 ^a^	118 ± 11.4 ^abc^	88 ± 7.6 ^abcd^	74 ± 6.4 ^abcd^	53 ± 5 ^bcdef^
	CHOL (mg/dL)	72 ± 6.7	61 ± 6.8 ^a^	63 ± 5.4 ^a^	158 ± 14.3 ^abc^	111 ± 9.2 ^abcd^	95± 8.4 ^abcde^	77 ± 7.6 ^bcdef^
	LDL-c (mg/dL)	35 ± 3.7	28 ± 3.4 ^a^	27 ± 3.8 ^a^	89 ± 7.1 ^abc^	62 ± 5.3 ^abcd^	48 ± 5.6 ^abcde^	33 ± 4.3 ^bcdef^
	HDL-c (mg/dL)	15 ± 2.8	22 ± 2.4 ^a^	25.6 ± 3.4 ^ab^	5.2 ± 0.67 ^abc^	9.8 ± 1.4 ^abcd^	12.3 ± 5.6 ^abcde^	16.5 ± 2.3 ^def^
Liver	TGs (mg/g)	4.4 ± 0.3	3.37 ± 0.4 ^a^	3.34 ± 0.4 ^ab^	8.8 ± 0.7 ^abc^	6.4 ± 0.8 ^abcd^	5.3 ± 0.5 ^abcde^	4.5 ± 2.3 ^bcdef^
	CHOL (mg/dL)	6.1 ± 0.6	5.1 ± 0.5 ^a^	0.49 ± 0.4 ^ab^	9.3 ± 1.1 ^abc^	8.1 ± 0.7 ^abcd^	7.2 ± 0.7 ^abcde^	5.9 ± 0.6 ^bcdef^
Stool	TGs (mg/g dry)	1.9 ± 0.23	1.8 ± 0.17	2.09 ± 0.22	5.7 ± 0.6 ^abc^	5.2 ± 0.5 ^abc^	5.8 ± 0.6 ^abc^	5.4 ± 0.4 ^abc^
	CHOL (mg/g dry)	2.4 ± 0.21	2.3 ± 0.3	2.5 ± 0.3	6.3 ± 0.8 ^abc^	5.9 ± 0.6 ^abc^	6.1 ± 0.7 ^abc^	6.4 ± 0.7 ^abc^

Data are presented as means ± SD (n = 8/group). The value of significance was determined at *p* < 0.05. a: significantly different as compared to the control group; b: significantly different as compared to the NAC-treated control rats; c: significantly different as compared to the γ-Oryzanol-treated rats; and d: significantly different as compared to the HFD-fed group; e: significantly different as compared to the HFD + NAC-treated rats; and f: significantly different as compared to the HFD + γ-Oryzanol-treated rats. TGs: triglycerides; CHOL: total cholesterol; LDL-c: low-density lipoprotein cholesterol; and HDL: high-density lipoprotein cholesterol.

## Data Availability

The datasets used and analyzed during the current study are available from the corresponding author upon reasonable request.
